# Should low-risk DCIS lose the cancer label? An evidence review

**DOI:** 10.1007/s10549-023-06934-y

**Published:** 2023-04-19

**Authors:** Tara Ma, Caitlin R. Semsarian, Alexandra Barratt, Lisa Parker, Nirmala Pathmanathan, Brooke Nickel, Katy J. L. Bell

**Affiliations:** 1 School of Public Health, The University of Sydney, Sydney, NSW 2006 Australia; 2 Wiser Healthcare, Sydney, Australia; 3 Sydney School of Pharmacy, Charles Perkins Centre, Faculty of Medicine and Health, University of Sydney, Sydney, Australia; 4 Department of Radiation Oncology, Royal North Shore Hospital, Sydney, Australia; 5 Western Sydney Local Health District, Sydney, Australia; 6 Westmead Breast Cancer Institute, Westmead Hospital, Sydney, Australia

**Keywords:** Ductal carcinoma in situ, Breast cancer, Overdiagnosis, Histopathology, Epidemiology

## Abstract

**Background:**

Population mammographic screening for breast cancer has led to large increases in the diagnosis and treatment of ductal carcinoma in situ (DCIS). Active surveillance has been proposed as a management strategy for low-risk DCIS to mitigate against potential overdiagnosis and overtreatment. However, clinicians and patients remain reluctant to choose active surveillance, even within a trial setting. Re-calibration of the diagnostic threshold for low-risk DCIS and/or use of a label that does not include the word ‘cancer’ might encourage the uptake of active surveillance and other conservative treatment options. We aimed to identify and collate relevant epidemiological evidence to inform further discussion on these ideas.

**Methods:**

We searched PubMed and EMBASE databases for low-risk DCIS studies in four categories: (1) natural history; (2) subclinical cancer found at autopsy; (3) diagnostic reproducibility (two or more pathologist interpretations at a single time point); and (4) diagnostic drift (two or more pathologist interpretations at different time points). Where we identified a pre-existing systematic review, the search was restricted to studies published after the inclusion period of the review. Two authors screened records, extracted data, and performed risk of bias assessment. We undertook a narrative synthesis of the included evidence within each category.

**Results:**

Natural History (*n* = 11): one systematic review and nine primary studies were included, but only five provided evidence on the prognosis of women with low-risk DCIS. These studies reported that women with low-risk DCIS had comparable outcomes whether or not they had surgery. The risk of invasive breast cancer in patients with low-risk DCIS ranged from 6.5% (7.5 years) to 10.8% (10 years). The risk of dying from breast cancer in patients with low-risk DCIS ranged from 1.2 to 2.2% (10 years). Subclinical cancer at autopsy (*n* = 1): one systematic review of 13 studies estimated the mean prevalence of subclinical in situ breast cancer to be 8.9%. Diagnostic reproducibility (*n* = 13): two systematic reviews and 11 primary studies found at most moderate agreement in differentiating low-grade DCIS from other diagnoses. Diagnostic drift: no studies found.

**Conclusion:**

Epidemiological evidence supports consideration of relabelling and/or recalibrating diagnostic thresholds for low-risk DCIS. Such diagnostic changes would need agreement on the definition of low-risk DCIS and improved diagnostic reproducibility.

**Supplementary Information:**

The online version contains supplementary material available at 10.1007/s10549-023-06934-y.

## Introduction

Widespread use of mammographic screening for breast cancer has dramatically increased the detection of early-stage breast cancers and precursor lesions, including ductal carcinoma in situ (DCIS). As some of these lesions are unlikely to progress to clinically significant disease during the person’s lifetime if left undetected and untreated, an important harm from mammography screening programmes is overdiagnosis and subsequent overtreatment [1–4]. Epidemiological trends in the United States (US) demonstrate increasing rates of breast cancer diagnoses alongside largely stable rates of metastatic disease and breast cancer mortality [5], observations consistent with a picture of overdiagnosis.

Those diagnosed with DCIS are usually offered surgery in the form of breast-conserving surgery or mastectomy. They may also undergo axillary lymph node intervention (sentinel lymph node biopsy and sometimes axillary lymph node clearance), radiotherapy, and endocrine therapies [6]. The risks of adverse outcomes and harms from at least some of these treatments may be acceptable trade-offs against the potential life-extending benefits for high-risk lesions [3]. However, in the case of low-risk DCIS, the trade-offs may no longer be acceptable, as there is much less potential for benefit. To prevent the potential for harm from overtreatment of low-risk lesions, active surveillance has been proposed as an alternative management strategy [7, 8]. There are several ongoing international clinical trials assessing the effects of active surveillance for low-risk DCIS compared to immediate treatment (LORETTA, LORD, LORIS, COMET) [1–3, 9, 10]. As there is not yet a consensus on what exactly constitutes low-risk DCIS, the trials have differing criteria, based on nuclear grade, patient age, and various clinical and pathological features. If evidence from these trials show that active surveillance is a safe and effective management option, then it may be offered in routine clinical practice, similar to what is now accepted practice for low-risk prostate cancer [11]. Recruitment into the trials has been slow, likely due to clinician and patient concerns about active surveillance as a management option [12–14]. Further, outside of trials currently only 3% of women diagnosed with DCIS in the US choose to forego both surgery and radiotherapy [3, 4]. To encourage uptake of active surveillance for low-risk DCIS where appropriate, consideration may be given to using alternative labels to describe such lesions that do not include the word ‘cancer’ [15, 16]. Alternatively, the DCIS label could be retained, but with a recalibration of diagnostic thresholds such that the DCIS term is only applied to lesions with a higher risk of adverse outcomes [17], and low-risk DCIS is given another label that does not contain the word ‘cancer’. These possibilities may encourage both clinicians and patients to choose active surveillance and other conservative management options where clinically appropriate [18–22].

The purpose of this review was to systematically appraise evidence that might support or refute the case for adopting alternative non-cancer labels and/or recalibration of diagnostic thresholds, for low-risk DCIS. We sought to understand (i) the natural history of low-risk DCIS if left untreated, (ii) the size of the “reservoir” of subclinical DCIS in people who died of other causes, (iii) the diagnostic reproducibility of DCIS, and (iv) whether there has been diagnostic drift that has expanded the DCIS definition over time, for example, where the same type of lesion would be diagnosed as cancer in recent years but would not have been in the past.

## Materials and methods

We searched for studies in four different categories: (1) natural history studies, including active surveillance and watchful waiting studies, (2) autopsy studies, (3) diagnostic reproducibility studies, and (4) diagnostic drift studies. PubMed and EMBASE databases were searched from inception to 8 October 2021. The search strategy is provided in Online Appendix 1. In addition, specific studies suggested by experts were also screened for potential inclusion, if they were determined to fit the inclusion criteria.

We identified an existing review for each of the natural history, autopsy, and reproducibility categories suggested by content experts, and a further review on reproducibility through initial literature searches. We therefore searched for additional studies published after the reviews’ inclusion period for these three categories. (See Online Appendix 2 for flow diagram.)

### Inclusion criteria

As the definition of what constitutes “low-risk” varies, we took a broad approach to inclusion and included all evidence relevant to DCIS without restriction on risk classification. As this study was focused on DCIS, we did not specifically include evidence about other types of borderline lesions, such as atypical ductal hyperplasia (ADH) or lobular carcinoma in situ (LCIS). In the natural history category, we included prospective or retrospective cohort studies where patients were diagnosed with DCIS but did not have local treatment (surgery or radiotherapy). In the autopsy category, we included autopsy studies reporting incidental (subclinical) breast cancer in individuals with no known history of breast cancer. In the diagnostic reproducibility category, we included cross-sectional studies with two or more independent diagnostic classifications of the same histopathological slides. In the diagnostic drift category, we included longitudinal studies with two or more independent diagnostic classifications of the same histopathological slides.

### Exclusion criteria

Abstracts, reviews, protocols of planned studies, and studies that were not in English were excluded. Studies where patients had only metastatic disease were also excluded. In the natural history category, studies which did not report on clinical outcomes relevant to disease progression (e.g. studies that only reported psychological outcomes) were excluded. In the autopsy category, studies only reporting cases of patients who were determined to have died of breast cancer that was not detected during life were excluded. In the diagnostic reproducibility and diagnostic drift categories, studies that reported only readings by non-pathologists, and studies that did not report on diagnostic classification, were excluded.

### Study selection, data extraction, quality assessment, and synthesis

The titles and abstracts were screened by one of the authors (CRS) and full text independently screened by two authors (TM and CRS). Discrepancies were resolved through discussion or through adjudication by two other authors (BN and KJLB). One of the authors (TM) extracted and summarized relevant study data in a spreadsheet that was developed and piloted by two other authors (BN and KJLB) and then assessed studies for risk of bias. Risk of bias was assessed using standardized tools adapted from ROBINS-I [23] for natural history studies, Hoy and colleagues’ tool [24] for autopsy studies, and QUADAS-2 [25] and QAREL [26] tools for diagnostic reproducibility and diagnostic drift studies. Both data extraction and quality assessment were checked by another author (BN or KJLB). Finally, a narrative synthesis of the evidence in each category was undertaken.

## Results

### Natural history studies (*n* = 11) (details in Table 1)

**Table 1:** Characteristics and key findings of the natural history studies that were included in this review

Authors	Study period	Country (Ethnicity)	Study design (Database used if relevant)	Sample size (no surgery)	Age (mean/median)	Grade or risk distribution	Mean/median follow-up	Summary of natural history findings	Risk of invasive breast cancer in untreated low-risk DCIS	Breast cancer mortality in untreated low-risk DCIS	Overall risk of bias ^a^
Erbas et al. ^b^	Up to 2006	N/A	Retrospective review including 4 studies	136	Not reported	Not reported	Not reported	The proportion of patients with DCIS misdiagnosed as benign who developed subsequent invasive cancer ranged from 14 to 53% between the four studies	Not reported	Not reported	High ^c^
Byng et al	1992–2016	US (77.9% white, 10.7% black)	Retrospective population database study (SEER database)	85,982 (1650)	40 +	17.5% Grade I; 44.6% Grade II; 38.0% Grade III	N/A	The prognosis of DCIS patients with low-risk features (Hispanic and non-Hispanic white women aged 50–69 at diagnosis, ER +, grade 1/2, ≤ 2 cm) who did not receive surgery remained comparable to their matched counterparts who had surgery	0.92% (95% CI 0.00–1.95%) at 5 years, 3.02% (95% CI 0.00–6.05%) at 10 years	Not reported	High
Akushevich et al	1992–2011	US (22.6% black in the active surveillance only group)	Retrospective population database study (SEER database)	22,567 (205)	65 +	> 30% ‘well differentiated’ or ‘moderately differentiated’	N/A	Active surveillance only was associated with higher risk of all-cause (HR 3.54; 95% CI 3.29–3.82) and breast cancer-specific (HR 10.73; 95% CI 8.63–13.35) mortality	Not reported	Not reported	High
Ryser et al	1992–2014	US (70.1% white, 14.9% black)	Retrospective population database study (SEER database)	1286 (1286)	60 (IQR 51–74)	Amongst patients with known tumour grade, 69.2% non-high grade	5.5 years (IQR 2.3–10.6)	111 (8.6%) developed ipsilateral invasive cancer. For grade I/II, the 10-year net risk of ipsilateral invasive cancer was 12.2% (95% CI 8.6%-17.1%) Amongst 239 patients with low-risk DCIS (non-high grade and ER or PR positive, diagnosed in women 40 years or older), ipsilateral invasive cancer was diagnosed in 6.5% (95% CI 3.5% to 12.0%) after 7.5-year follow-up	6.5% (95% CI 3.5%–12.0%) after 7.5 years	Not reported	High
Sagara et al	1988–2011	US (78.2% white, 15.2% black amongst non-surgery patients)	Retrospective population database study (SEER database)	57,222 (1169)	Not reported	Amongst 1169 without surgery, 192 low grade, 441 intermediate grade, 536 high grade	72 months	For low-grade DCIS, there was no significant difference for breast cancer-specific survival between the surgery and non-surgery groups	Not reported	Weighted 10-year breast cancer-specific survival was 98.8% at 10 years	High
Mannu et al	1988–2014	UK	Retrospective population database study (NHS breast screening programme)	35,024 (1452)	Range from < 55 to > = 65	35.1% low -intermediate grade, 51.5% high grade, 13.4% unknown grade	Not reported	The observed to expected ratio of invasive cancer 3 years from DCIS diagnosis was 2.63 (95% CI 2.47–2.81) for women who received surgery and 3.38 (2.70–4.24) for no surgery	Not reported	Not reported	High
Co et al	1997–2006	Hong Kong, China	Retrospective population database study (Hong Kong Cancer Registry)	1391 (280)	49.2 (Range 30–70)	372 ‘low risk’; 777 ‘high risk’	11.2 years	For low-risk DCIS (patients aged 46 years or above, low/intermediate nuclear grade), there was no statistically significant difference in incidence of subsequent invasive cancer with or without surgery	716 per 100 000 person years	10-year breast cancer-specific survival was 97.8%)	High
Maxwell et al	1996–2016	UK	Retrospective (patients identified via cancer registries, NHS programmes and clinical studies)	89 (89)	75 (Range 44–94)	29 high grade, 31 intermediate, 17 low grade, 12 unknown	59 months	29/89 (33%) of AS cases developed invasive cancer. Incidence of invasive cancer was higher in high-grade DCIS. (Only 3/17 (18%) with low-grade DCIS developed invasive cancer)	3/17 (18%) after median 51 months	Not reported	High
Grimm et al	2009–2014	US (62% white or Caucasian; 17% African American)	Retrospective review of clinical records	29 (29)	Non-trial: 61.1 (Range 40.8–88.8); Letrozole trial: 62.5 (Range 50.8–70.6)	1 low grade, 14 intermediate grade, 13 high grade, 1 unknown	2.7 years (non-trial)	3 out of 29 AS cases developed invasive cancer	0/1 (0%)	0/1 (0%)	High
Meyerson et al	2003–2008	US	Retrospective review of clinical records	14 (14)	49 (Range 41–71)	4 low grade, 8 intermediate grade, 2 high grade	42.3 months	5 out of 14 AS cases developed invasive cancer	1/4 (25%) after 10.1 months	Not reported	High

*AS* active surveillance

^a^See Online Appendix 3 for complete risk of bias assessment

^b^Review including four different cohorts

^c^Primary studies reported in the review were retrospective

One review was pre-identified in the natural history category and included. We screened titles and abstracts of 167 articles retrieved from the database search and two suggested by experts, reviewed the full text of 42 articles, and included 9 primary studies. The screening process is shown in Online Appendix 2a. Six of the primary studies were population database studies. Three studies were based on clinical records, and at least some patients received endocrine therapy in all these studies. All primary studies described in this section were retrospective studies and were assessed as having a high risk of bias overall.

The review by Erbas et al. [27] identified four studies that retrospectively examined cases of breast biopsies originally diagnosed as benign, to find cases of DCIS misdiagnosed as benign, and where follow-up data were available. Amongst the four studies, with a total of 136 cases (range 13–80 cases), the rate of development of invasive cancer (ipsilateral or contralateral) was 14–53%, after 1–31-year follow-up. No data were provided on low-risk DCIS specifically. No risk of bias assessments were reported by the reviewers.

Six population-based studies reported on evidence on DCIS outcomes from administrative databases. Four US studies utilized data from the National Cancer Institute’s surveillance, epidemiology, and end results (SEER) programme. Byng et al. [28] reported a propensity score-based analysis for 1,650 patients with DCIS managed without surgery or radiotherapy (364 grade I, 786 grade II, and 500 grade III) amongst a total 85,982 patients with DCIS. They found that patients with low-risk DCIS had a similar risk of invasive breast cancer at long-term follow-up whether they received treatment or not (low risk defined as Hispanic or non-Hispanic white ethnicity, age 50–69 at diagnosis, oestrogen receptor positive, Grade I or II, lesion size < 2 cm). The risk of ipsilateral invasive breast cancer in the untreated low-risk DCIS group was estimated to be 0.92% (95% CI 0.00–1.95%) at 5 years and 3.02% (95% CI 0.00–6.05%) at 10 years. The combined risk of contralateral and ipsilateral invasive cancer for women with untreated low-risk DCIS was within the 10-year population-wide age-specific risk of invasive breast cancer for US women. In another propensity score-based analysis, Akushevich et al. [29] compared outcomes for women aged 65 + diagnosed with DCIS who (i) had treatment within a year of diagnosis before any evidence of cancer progression (*n* = 21,772); (ii) had treatment within a year of diagnosis after evidence of cancer progression (*n* = 431); and (iii) did not have treatment within a year of diagnosis (*n* = 405). They found that patients who did not have treatment within a year of diagnosis (group iii) had a higher risk of all-cause (HR 3.54; 95% CI 3.29–3.82) and breast cancer-specific (HR 10.73; 95% CI 8.63–13.35) mortality compared with those who received treatment within a year of diagnosis (either before or after evidence of cancer progression). However, results for low-risk DCIS were not reported. Ryser et al. [30] found that amongst 1286 DCIS patients who did not have local treatment, 111 patients (8.6%) were diagnosed with ipsilateral invasive cancer after a median 5.5 years of follow-up. Amongst the 239 patients with low-risk DCIS (non-high grade and ER or PR positive diagnosed in women 40 years or older), the cumulative net risk of ipsilateral invasive cancer was 6.5% (95% CI 3.5–12.0%) after 7.5-year follow-up. Sagara et al. [31] reported outcomes on 1169 patients with DCIS managed without surgery amongst a total of 57,222 patients with DCIS. They found that the degree of survival benefit conferred by surgery differed by nuclear grade (*p* = 0.003), after adjusting for other relevant patient characteristics. For low nuclear-grade DCIS, there was no significant difference in the weighted 10-year breast cancer-specific survival between the surgery and non-surgery groups (98.6% and 98.8%, respectively, *p* = 0.95).

The UK study by Mannu et al. [32] reported outcomes for 1452 patients with DCIS managed without surgery amongst a total of 35,024 patients with DCIS. They found that compared with women in the general population, women with screen-detected DCIS (whether they had surgery or not) had a higher long-term risk of developing invasive breast cancer (ipsilateral or contralateral), for at least 20 years after diagnosis. At 20 years after diagnosis, the cumulative risk of invasive breast cancer was 15.6%, and the cumulative risk of death from breast cancer was 3.8%. The authors also found that women with DCIS who did not have surgery had higher rates of invasive cancer and breast cancer mortality, compared with those who had surgery. The observed to expected ratio for death caused by breast cancer was 1.72 (1.38 to 2.12) for women who had received surgery and 3.89 (2.31 to 6.57) for women who had not received surgery. However, results for low-risk DCIS were not reported. The Hong Kong study by Co et al. [33] reported on 280 patients with DCIS managed without surgery amongst a total 1391 patients, utilizing the local cancer registry. They found that 10-year breast cancer-specific survival was 97.8% for low-risk DCIS without surgery (patients aged 46–70 years with low or intermediate nuclear-grade DCIS). The incidence of subsequent invasive cancer was similar for patients with low-risk DCIS for those who had surgery (989.4 per 100,000) and those who did not (716 per 100 000 person years; *p* = 0.64).

Three retrospective cohort studies reported on evidence on DCIS outcomes from clinic databases. The UK study by Maxwell et al. [34] reported on 89 patients with DCIS, including 17 with low-grade DCIS, who did not have surgical treatment as identified via cancer registries, NHS programmes, and clinical studies. Overall, 29 cases (33%) were diagnosed with invasive cancer, after a median of 3 years and 9 months follow-up. Amongst 17 patients with low-grade DCIS, 3 (18%) were diagnosed with invasive cancer, after a median of 4 years and 3 months. The US study by Grimm et al. [35] reported on follow-up of 29 patients, including one case of low-grade DCIS who did not develop invasive cancer. The US study by Meyerson et al. [36] reported on 14 patients with DCIS, including 4 with low-grade DCIS. Five patients, including one with low-grade DCIS, were diagnosed with invasive cancer, whilst the other 9 remained free of invasive cancer after a median follow-up of 2 years and 7.8 months.

The details of individual studies included in this section are listed in Table 1. All the primary studies identified in this category were assessed to be at high risk of bias overall. The risk of bias assessment for each study is available in Appendix 3a.

### Autopsy studies (*n* = 1)

For autopsy studies we pre-identified a systematic review by Thomas et al. [37]. We retrieved 73 articles from the literature search, published after 8 April 2016 (the cut-off date for the Thomas review). After title and abstract screening, 5 articles were retained for full-text screening, but none of these fulfilled the inclusion criteria. Therefore, only the evidence from the pre-identified systematic review was included. The screening process is shown in Online Appendix 2b.

The systematic review included 13 studies providing 14 datasets, published from 1954 to 2015. It included a total of 2363 autopsies with 99 cases of incidental subclinical breast cancer or precursor lesions, from Denmark, Italy, Canada, the USA, Chile, Japan, Ghana, Australia, and Norway. The mean prevalence of ‘in situ cancer’, including DCIS and lobular carcinoma in situ (LCIS), was 4.5% (range 0–18.7%), whilst the mean prevalence of invasive cancer was 1.5% (range 0–7.1%). However, studies which conducted more thorough pathology examinations yielded greater prevalence of subclinical in situ cancer. After modelled adjustment for the less thorough studies, the mean prevalence of subclinical in situ cancer was estimated to be 8.9%. There was no statistically significant trend in the overall prevalence of breast cancer and its precursor lesions over time, nor with age.

### Diagnostic reproducibility studies (***n*** = 13) (details in Table 2)

**Table 2:** Characteristics of the diagnostic reproducibility studies that were included in this review

Authors	Year	Country	Type of pathologists	Sample, pathologists (N) cases (n)	Included borderline lesion type	Diagnostic categories assessed	Reference standard (if used)	Interobserver agreement	Intraobserver agreement	Overall risk of bias^a^
Segnan et al.^b^	2017	Multiple^b^	Varies by primary study	Total *N* = unclear; *n* = 13,017 (range 12 to 2004 per study) across 27 studies	DCIS	Invasive breast cancer, DCIS, benign lesions	Varies by primary study	DCIS misclassified as invasive on the total of malignant (DCIS and invasive) lesions range from 0 to 12% amongst those reported	N/A	Varies by primary study (reported in review)
Van Bockstal et al.^b^	2020	Multiple^b^	Varies by primary study	Total *N* = 165 (range 2 to 39 per study); *n* = 1301 (range 23 to 350 per study) across 12 studies	DCIS	DCIS	Varies by primary study	*k* ranges from 0.23 to 0.78 in different parameters of grading in different studies (although not directly comparable between studies). Most k-values are < 0.60	N/A	Not reported in review
Mercan et al.	2019	US	Pathologists who regularly interpreted breast biopsy specimens	*N* = 87, *n* = 240 (but each pathologist only read 60 slides)	DCIS, atypia	Benign without atypia, atypia, DCIS, and invasive cancer	Expert panel consensus	N/A	Invasive vs non-invasive: 0.84 sensitivity, 0.99 specificity; DCIS vs atypia: 0.70 sensitivity, 0.82 specificity; Benign vs DCIS or atypia: 0.72 sensitivity, 0.62 specificity	Low
Qiu et al.	2019	US	Pathologists who routinely practice breast pathology	*N* = 3, *n* = 58	Papilloma with ADH, papilloma with DCIS	Benign papilloma; papilloma with atypia and/or DCIS; invasive carcinoma with papillary features	N/A	Overall *k* = 0.79, 86% agreement; papilloma with atypia/DCIS *k* = 0.74, 84% agreement	N/A	Moderate
Brunye et al.	2017	US	Pathologists varied in training and experience interpreting breast pathology	*N* = 40, *n* = 24 (but each pathologist read 12)	DCIS, atypia	Benign without atypia, atypia, DCIS, and invasive cancer	Expert panel consensus	DCIS: 52% concordance with consensus, 17% ‘above consensus diagnosis’, 31% ‘below consensus diagnosis’	N/A	Moderate
Jackson et al.	2017	US	Pathologists who had interpreted breast specimens in the prior year and planned to continue in the next year	*N* = 49, *n* = 240 (but each pathologist only read 60 slides)	DCIS, atypia	Benign without atypia, atypia, DCIS, and invasive cancer	Expert panel consensus	DCIS: 79% (95% CI 76–81%)	DCIS: 84% (95% CI 81–87%)	Low
Tozbikian et al.	2017	US	Pathologists who specialize in breast pathology	*N* = 5, *n* = 105	Borderline ADH/DCIS lesions	1 = benign, 2 = ADH, or 3 = DCIS	Majority diagnosis of > = 3 pathologists if available	There was agreement by 5/5 reviewers in 32/105 cases (30%); 4/5 reviewers in 44/105 cases (42%); 3/5 reviewers in 26/105 cases (25%)	N/A	Low
Trocchi et al.	2017	Germany	Experienced breast pathologists	*N* = 2, *n* = 256	B3, B4, B5a (in situ)	B-categorization scheme: B1: normal or not interpretable, B2: benign, B3: benign but of uncertain biological potential, B4: suspicious of malignancy, B5: malignant	N/A	N/A	Chance-corrected observed agreement between the first and second assessments was *k* = 0.85 (95% CI: 0.80–0.89) for pathologist 1 and *k* = 0.81 (95% CI 0.76–0.87) for pathologist 2	Moderate
Rakha et al.	2017	UK	Pathologists who report breast pathology (including breast specialists and general pathologists)	*N* = 536–675, *n* = 240	DCIS, atypia	Benign without atypia, atypia, DCIS, and invasive cancer	Not clearly specified	205/240 (85.4%) showed < 5% disconcordance overall; *k* = 0.88 for DCIS diagnosis	N/A	Moderate
Rakha et al.	2016	UK	Pathologists who report breast pathology (including breast specialists and general pathologists)	*N* = not specified (up to > 600), *n* = 6	Papillary DCIS	Papillary DCIS, encapsulated PC, solid PC, and invasive PC	Not clearly specified	Diagnoses of malignancy was 88% for papillary DCIS. 77% correctly classified papillary DCIS as in situ. 28% misclassified invasive PC cases as in situ	N/A	Moderate
Van Seijen et al.	2021	UK, US, Netherlands	Three breast pathologists from each country	*N* = 9, *n* = 425	DCIS	DCIS grade (1, 2, or 3) and a range of histological features used for grading	N/A	Moderate agreement (*k* = 0.50) on DCIS grading. Substantial diagnostic variation in various histological features (*k* = 0.33–0.61)	N/A	Moderate
Tsuda et al	2021	Japan	Not specified	*N* = 24 to 29; *n* = 46	DCIS	Nuclear grade, comedo necrosis	N/A	Interobserver agreement of nuclear grade was moderate (*k* = 0.595 and 0.519). Interobserver agreement of comedo necrosis was substantial (*k* = 0.753)	N/A	Moderate
Onega et al.	2017	US	Pathologists who had interpreted breast specimens in the prior year and planned to continue in the next year	*N* = 115, *n* = 73	DCIS	High-grade DCIS, low-grade DCIS	Expert panel consensus	Agreement with reference standard was 46% (95% CI 42–51%) for low-grade DCIS and 83% (95% CI 81–86%) for high-grade DCIS	N/A	Low

*ADH* atypical ductal hyperplasia, *DCIS* ductal carcinoma in situ, *PC* papillary carcinoma

^a^See Online Appendix 3 for complete risk of bias assessment

^b^Systematic review including evidence from multiple primary studies

Two reviews and one primary study suggested by experts were included in the reproducibility category. We screened titles and abstracts of 221 articles, reviewed the full text of 57, and included a further 10 primary studies. Therefore, we reviewed evidence from 2 reviews and 11 primary studies. The screening process is shown in Online Appendix 2c.

The review by Segnan et al. [38], which focused on the reproducibility of the diagnosis of breast lesions as benign, DCIS, or invasive cancer, included 27 studies of diagnostic reproducibility, with a total of 13,017 lesions. It found that there was a high level of heterogeneity between studies, and overall there was a non-negligible false-positive rate. Amongst 6 studies with consecutive, random, or stratified samples, 0 to 3.2% of benign lesions were misclassified as DCIS. Amongst 5 studies with only cases selected for a second opinion, 0 to 34.48% of benign lesions were misclassified as DCIS. Amongst five studies with enriched samples, 0 to 9.64% of benign lesions were misclassified as DCIS. Of the included studies, the US study by Elmore et al. [39] is notable for having both a large number of lesions examined (*n* = 240) and a large number of readers (*n* = 115 + 3). In this study, there was 75.3% (95% CI 73.4–77.0%) overall agreement amongst the readers regarding the diagnosis of breast lesions as benign without atypia, atypia, DCIS, or invasive cancer. However, benign lesions without atypia were misclassified as DCIS 2.22% of the time and lesions with atypia were misclassified as DCIS 17.1% of the time [39].

The review by Van Bockstal et al. [40], which focused on the reproducibility of the grading of DCIS lesions, included 12 studies of diagnostic reproducibility with a total of 1301 lesions. It found that all studies showed substantial interobserver variability in the assessment of parameters used in different grading systems, including nuclear grade, presence of comedo necrosis, and DCIS growth patterns. The kappa values for these parameters ranged from 0.23 (fair agreement) to 0.78 (substantial agreement), with most values < 0.60 (i.e. moderate agreement at best). The authors suggested that two-tier histopathologic assessment (i.e. classifying DCIS into two-grade categories rather than three), the implementation of digital pathology and deep learning algorithms and additional immunohistochemical and molecular testing, might be able to improve reproducibility.

In the new primary studies we identified, 8 studies reported on the differentiation between DCIS and other diagnoses. The US study by Mercan et al. [41] found that, in their sample of pathologists, the sensitivity and specificity for the differentiation of DCIS vs atypia was 0.70 and 0.82, respectively. The US study by Qiu et al. [42] found 84% agreement for diagnosis of the category ‘papilloma with atypical ductal hyperplasia or DCIS’ (*k* = 0.74). The US study by Brunye et al. [43] found that amongst cases with a consensus diagnosis of DCIS, there was only 52% mean concordance with the consensus, with 17% providing an ‘above consensus diagnosis’ and 31% a ‘below consensus diagnosis’. The US study by Jackson et al. [44] found 79% (95% CI 76–81%) interobserver agreement and 84% (95% CI 81–87%) intraobserver agreement for the diagnosis of DCIS. The US study by Tozbikian et al. [45], in which five pathologists who specialized in breast pathology were asked to diagnose borderline atypical ductal hyperplasia (ADH)/DCIS lesions as either benign, ADH, or DCIS, found total agreement in only 30% of cases. The German study by Trocchi et al. [46], which assessed intraobserver agreement in two experienced breast pathologists, found almost perfect agreement between the first and second assessments (chance-corrected *k* > 0.8 for both pathologists), when using the B-categorization scheme. The UK study by Rakha et al. [47], reporting results from the NHS Breast Screening Programme external quality assessment scheme, found a high level of reproducibility in the diagnosis of DCIS (*k* = 0.88). The 2016 UK study by Rakha et al. [48], reporting from the same programme, found that 28% of pathologists had misclassified cases of invasive papillary breast cancer as being in situ, whilst 11% of pathologists had misclassified a single case of papillary DCIS as benign or atypia.

Three of the new studies reported on the grading of DCIS cases. Similar to the Van Bockstal review, all three studies found only limited agreement amongst pathologists. The study by Van Seijen et al. [49], involving breast pathologists from the UK, the US, and the Netherlands, also found only moderate agreement (*k* = 0.50) on DCIS grading. It also found sub-optimal diagnostic reproducibility of various histological features, including necrosis, calcifications, lymphocytic infiltrate, periductal fibrosis, mitoses, and architectural pattern (*k* = 0.33 to 0.61). The Japanese study by Tsuda et al. [50] found that interobserver agreement of nuclear grade was moderate (*k* < 0.6). However, interobserver agreement of comedo necrosis was substantial (*k* = 0.753). The US study by Onega et al. [51] found that agreement with the reference standard diagnosis was 46% (95% CI 42–51%) for low-grade DCIS and 83% (95% CI 81–86%) for high-grade DCIS. Low-grade DCIS cases were diagnosed as high-grade or invasive cancer 23% of the time and diagnosed as benign or atypia 30% of the time.

The details of individual studies included in this section are listed in Table 2. All primary studies in this section were rated as having either low or moderate risk of bias. The risk of bias assessment for each study is available in Appendix 3b.

### Diagnostic drift studies (*n* = 0)

No relevant reviews were identified for this category. We retrieved 254 articles from the literature search. After title and abstract screening, 39 articles were retained for full-text screening, but none of these fulfilled the inclusion criteria. The screening process is shown in Online Appendix 2d.

## Discussion

This review found several lines of evidence relevant to the consideration of relabelling low-risk DCIS and/or recalibrating the diagnostic threshold of DCIS. Whilst there have been no completed prospective active surveillance trials, evidence about the natural history of DCIS is available from retrospective database reviews and retrospective cohort studies. Overall, DCIS is associated with a non-negligible risk of invasive cancer and breast cancer death if untreated; however, this appears to be lower for low-risk DCIS. It is possible that the low-risk lesions may be a risk indicator for invasive cancer rather than a direct precursor lesion. This is supported by our finding of similar outcomes on long-term follow-up of low-risk DCIS whether or not they were treated with surgery. The autopsy studies demonstrate a reservoir of subclinical in situ breast cancer that had not caused symptoms or contributed to the women’s deaths. Together, these two lines of evidence support consideration of offering active surveillance to manage low-risk DCIS. The diagnostic reproducibility evidence is mixed, and there is clearly sub-optimal agreement in the grading of DCIS. Finally, we found no evidence regarding diagnostic drift.

A major issue that needs to be resolved before relabelling low-risk DCIS and/or recalibrating the diagnostic threshold of DCIS can potentially occur is the current lack of agreement on how low-risk DCIS is to be defined. The natural history studies we found used different definitions, as do the trials that are currently underway. The diagnostic reproducibility of DCIS grading, which is part of the criteria used to classify DCIS as low risk, is also sub-optimal. If low-risk DCIS is to become a separate diagnostic category with different treatment recommendations, there will need to be an agreed upon definition for what constitutes a low-risk lesion, and the diagnostic reproducibility of such lesions must be improved.

The results of this review are consistent with the understanding that screening for breast cancer leads to overdiagnosis and overtreatment, i.e. the diagnosis and treatment of lesions that would remain clinically insignificant [16, 17, 52]. The extent overdiagnosis has been under-appreciated [15]. In particular, although DCIS was rarely diagnosed before screening, it is now routinely treated with aggressive therapy, despite uncertainty about its natural history [6], including whether some cases are already metastatic by the time they are detectable [53]. At the population level, aggressive treatment of DCIS has not led to a drop in the incidence of invasive cancer [15, 17] or metastatic cancer [53]. At the individual level, a recent RCT found that residual DCIS (left after surgery) appeared to make little difference to the effect of neoadjuvant chemotherapy on risk of cancer recurrence [54]. In recent years, active surveillance has been proposed as an alternative for low-risk DCIS. However, there remains a perception that aggressive treatment is required for cancer, and patients are often hesitant to choose active surveillance [16]. This has led to proposals for relabelling low-grade DCIS using terminology that does not include the word carcinoma, to reflect its indolent nature, and to encourage the adoption of less aggressive treatment options [15–17, 55]. Terms like “indolent lesions of epithelial origin” (IDLE) [17] and “ductal intraepithelial neoplasia” (DIN) [56] have been proposed. It has been shown that, in a hypothetical scenario, more women preferred surgical treatment when DCIS was described as a cancer, compared to describing it as a ‘breast lesion’ or ‘abnormal cells’ [16].

Overdiagnosis and overtreatment of DCIS are complex problems and a multi-pronged strategy will be needed to wind back the harms resulting from them. Changing the terminology used to describe low-risk lesions may form part of the overall strategy, but is unlikely to solve these problems on its own. If terminology change is thought to have merit, in order for this to be implemented there would first need to be an agreement on what is classified as low risk. Widespread mammography screening has unearthed a spectrum of subclinical breast lesions, which exist across a continuum of risk [57]. As with the detection of other asymptomatic conditions, choosing the threshold for dichotomising into low-risk versus high-risk categories is fraught. The increasing availability of molecular indicators [57–61] and artificial intelligence prediction of clinically aggressive behaviour [62, 63] may help with this determination. There is also the further issue that even lesions classified low risk and not labelled ‘cancer’ may be overtreated, as seen by ADH overtreatment [64, 65]. This underlies the importance of clear communication about the risk of adverse outcomes in each case, regardless of the diagnostic label used [66]. There also needs to be a more sophisticated understanding of the broad clinical spectrum of disease for breast lesions detected using modern screening technology.

The strengths of this review lie in its methodological rigour, including a comprehensive search of the literature supplemented by articles suggested by experts in the field, and a critical appraisal of included studies. We present evidence on DCIS overall, as well as low-risk DCIS where this was reported. This is also the first review of the evidence with the aim of answering the question of whether low-risk DCIS should become a separate diagnostic category with a non-cancer diagnostic label. The limitations of this review include the high risk of bias and inadequate reporting in many of the included studies, with a relative lack of data on low-risk DCIS specifically. Those studies that did report on low-risk lesions differed in the criteria they used to define this. No data from active surveillance trials are available yet, nor any other prospective cohort studies. Some of the database studies did not report the risk of developing invasive cancer after diagnosis of low-risk DCIS, which is a patient-relevant outcome. However, since this outcome is also subject to overdiagnosis through diagnostic scrutiny, it may be less reliable as a prognostic indicator than breast cancer-specific mortality. We also found no studies on diagnostic drift. Diagnostic drift refers to changes in the criteria used for diagnostic classification over time and includes both explicit definition changes, e.g. definition changes in newer editions of the WHO Blue Book, and implicit changes in the definition used by pathologists to categorize lesions, which are not formally documented. There is an important gap in the evidence base here.

Randomized trials currently underway will generate definitive evidence on the safety of active surveillance [1–3, 9, 10, 67], but these data will not be available for some years. Meanwhile, the evidence summarized in this review may facilitate opening discussion about the benefits and harms of removal of “cancer” from the diagnostic label of low-risk DCIS. When the evidence from the trials become available, they will further inform the debate, including a more precise definition for low-risk DCIS that has wide acceptance in clinical and pathology communities.

## Supplementary Information

Below is the link to the electronic supplementary material.

Supplementary file1 (DOCX 1132 kb)

## Data Availability

Enquiries about data availability should be directed to the authors.
