# Supplementary material for: Should low-risk DCIS lose the cancer label? An evidence review
Source: Breast Cancer Res Treat. 2023 Apr 19;199(3):415–33. doi: 10.1007/s10549-023-06934-y (PMC10175360; doi:10.1007/s10549-023-06934-y)
Supplement: Supplementary file 1 — Supplementary file1 (DOCX 1132 kb) [file 10549_2023_6934_MOESM1_ESM.docx]

Online Appendix

Includes:

Online Appendix 1: Search strategies

Online Appendix 2: Literature search and study selection process

Online Appendix 3: Risk of bias assessment

Online Appendix 1: Search strategies

## 1a. Natural history

*EMBASE*

‘active surveillance’.mp AND (breast.mp OR DCIS.mp OR ‘ductal carcinoma in situ’.mp OR mammogram*.mp OR mammar*.mp), limit to *human* and *article*

*PubMed*

"active surveillance"[Text Word] AND (breast[Text Word] OR DCIS[Text Word] OR "ductal carcinoma in situ"[Text Word] OR mammogra*[Text Word] OR mammar*[Text Word]), limit to *humans* and *journal article*

## 1b. Autopsy studies

*EMBASE*

((autopsy.mp OR autopsies.mp OR necropsies.mp) AND (population.mp OR study.mp OR series.mp)) AND (incidental.mp OR incidence.mp OR prevalence.mp OR latent.mp OR frequency.mp) AND (breast.mp OR DCIS.mp OR ‘ductal carcinoma in situ’.mp), limit to *human* and *article* and limit to dd=20160409-20230101 OR rd=20160409-20230101

*PubMed*

((autopsy[Text Word] OR autopsies[Text Word] OR necropsies[Text Word]) AND (population[Text Word] OR study[Text Word] OR series[Text Word])) AND (incidental[Text Word] OR incidence[Text Word] OR prevalence[Text Word] OR latent[Text Word] OR frequency[Text Word]) AND (DCIS[Text Word] OR breast[Text Word] OR "ductal carcinoma in situ"[Text Word]), limit to *humans*, *journal article* and from 2016/04/09

## 1c. Reproducibility

*EMBASE*

(‘observer variation’/exp OR reproducibility/exp OR ‘diagnostic accuracy’/exp) AND (reproducibility.mp OR interobserver.mp OR inter-observer.mp OR observer.mp OR inter-institutional.mp OR ‘diagnostic agreement’.mp OR ‘diagnostic concordance’.mp OR ‘second opinion’.mp OR ‘second-opinion’.mp OR ((diagnosis.mp OR case.mp OR review.mp OR assessment.mp OR reporting.mp) AND (consistency.mp OR discrepancy.mp OR accuracy.mp))) AND (histopatholog*.mp OR histomorpholog*.mp OR histolog*.mp OR histoprognostic.mp OR pathology.mp) AND pathologist*.mp AND (breast.mp OR DCIS.mp OR ‘ductal carcinoma in situ’.mp), limit to *human* and *article* and limit to dd=20151031-20230101 OR rd=20151031-20230101

*PubMed*

("observer variation"[Mesh] OR "reproducibility of results"[Mesh]) AND (reproducibility[Text Word] OR interobserver[Text Word] OR inter-observer[Text Word] OR observer[Text Word] OR inter-institutional[Text Word] OR "diagnostic agreement"[Text Word] OR "diagnostic concordance"[Text Word] OR "second opinion"[Text Word] OR "second-opinion"[Text Word] OR ((diagnosis[Text Word] OR case[Text Word] OR review[Text Word] OR assessment[Text Word] OR reporting[Text Word]) AND (consistency[Text Word] OR discrepancy[Text Word] OR accuracy[Text Word]))) AND (histopatholog*[Text Word] OR histomorpholog*[Text Word] OR histolog*[Text Word] OR histoprognostic[Text Word] or pathology[Text Word]) AND pathologist*[Text Word] AND (breast[Text Word] OR DCIS[Text Word] OR "ductal carcinoma in situ"[Text Word]), limit to *humans*, *journal article* and from 2015/10/31

## 1d. Diagnostic drift

*EMBASE*

(‘diagnostic drift’.mp OR ‘stage migration’.mp OR ‘grade inflation’.mp OR (‘Will Rogers’.mp AND (effect.mp OR phenomenon.mp)) OR ((re-evaluate.mp OR reclassify.mp OR reclassification.mp OR application.mp) AND (cases.mp OR criteria.mp))) AND (breast.mp OR DCIS.mp or ‘ductal carcinoma in situ’.mp), limit to *human* and *article*

*PubMed*

("diagnostic drift"[Text Word] OR "stage migration"[Text Word] OR "grade inflation"[Text Word] OR ("Will Rogers"[Text Word] AND (effect[Text Word] OR phenomenon[Text Word])) OR ((re-evaluate[Text Word] OR reclassify[Text Word] OR reclassification[Text Word]) AND cases[Text Word])) AND (breast[Text Word] OR DCIS[Text Word] OR "ductal carcinoma in situ"[Text Word]), limit to *humans* and *journal article*

Online Appendix 2: Literature search and study selection process

2a. Natural history


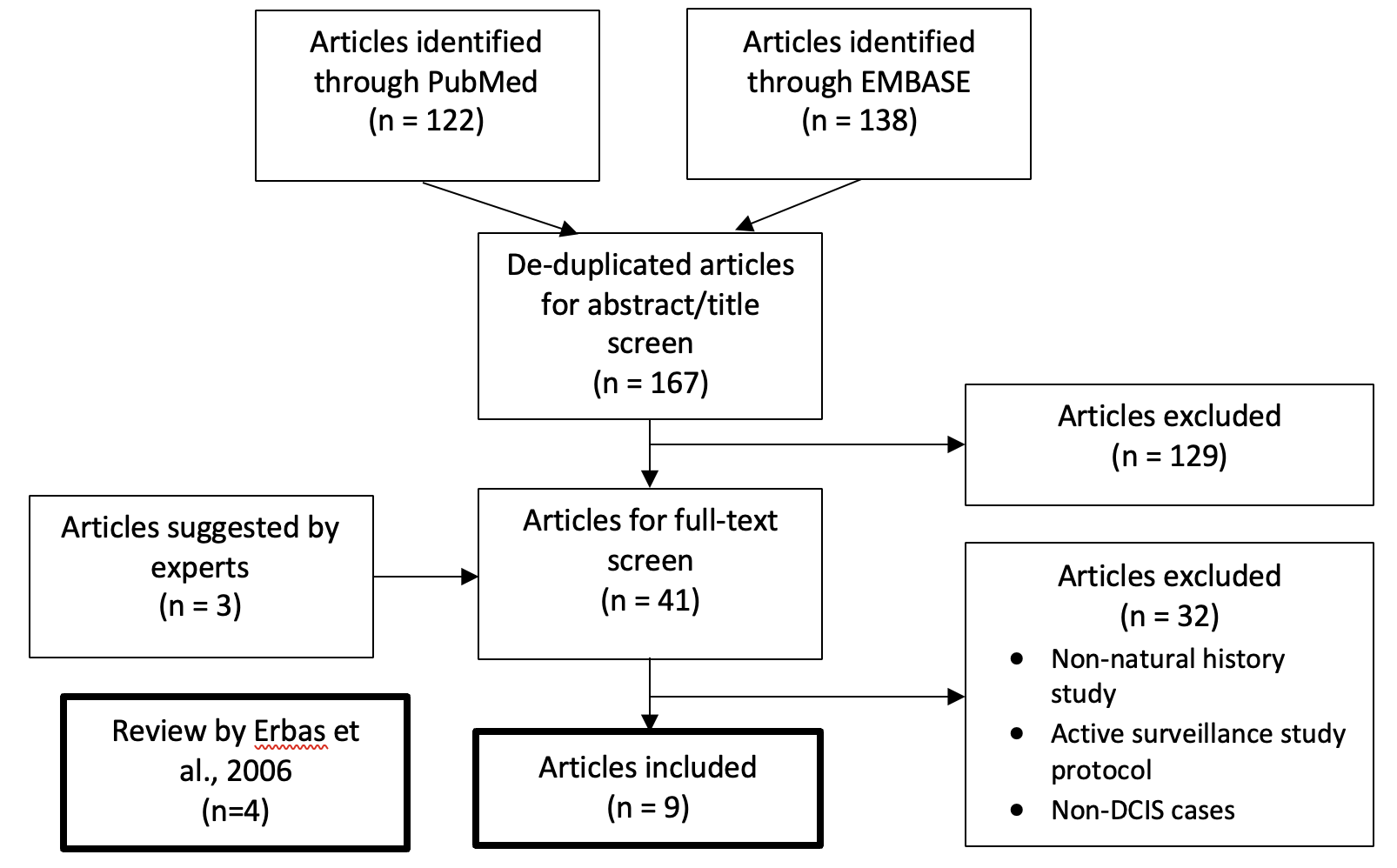


2b. Autopsy studies


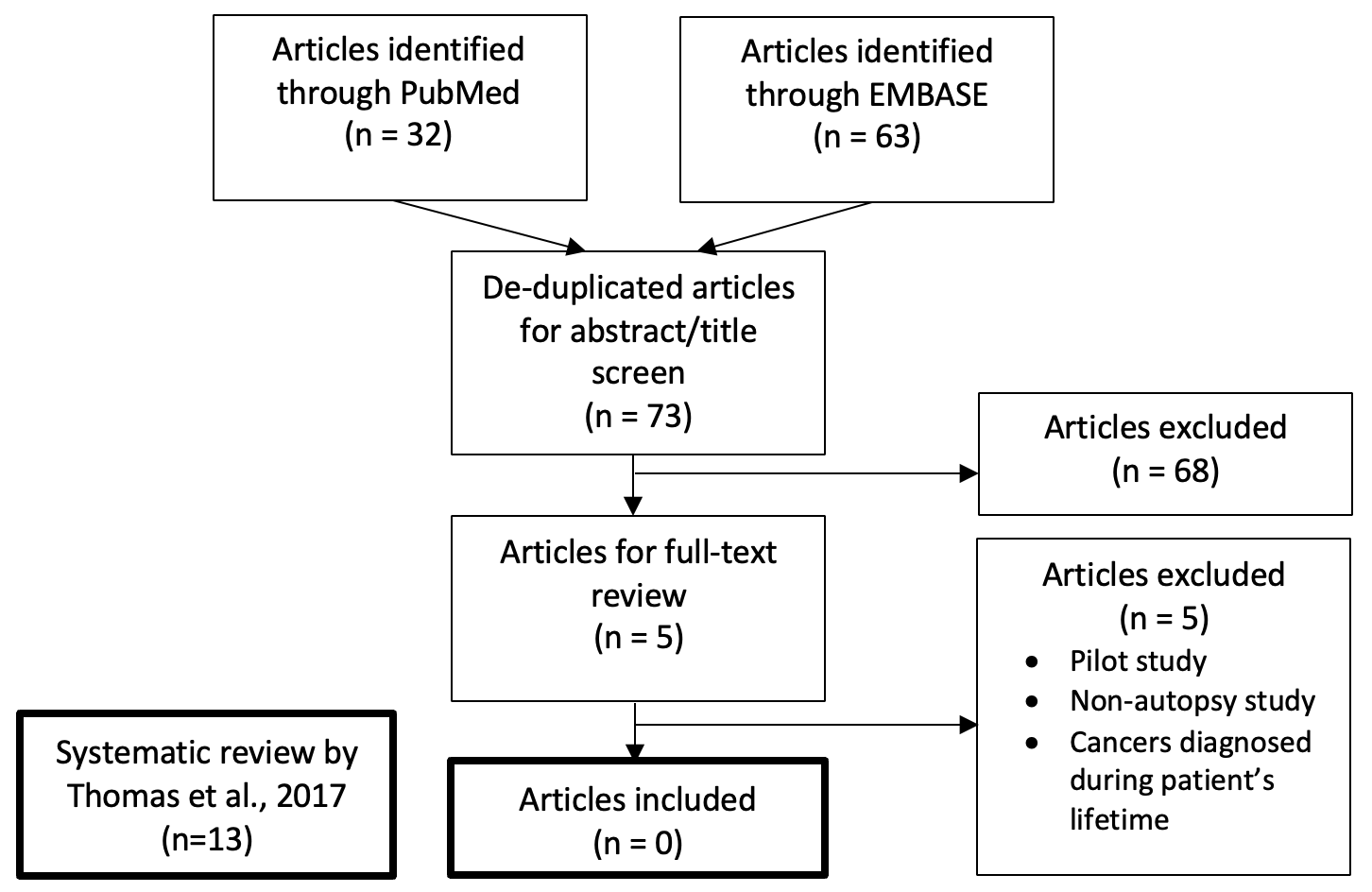


2c. Reproducibility


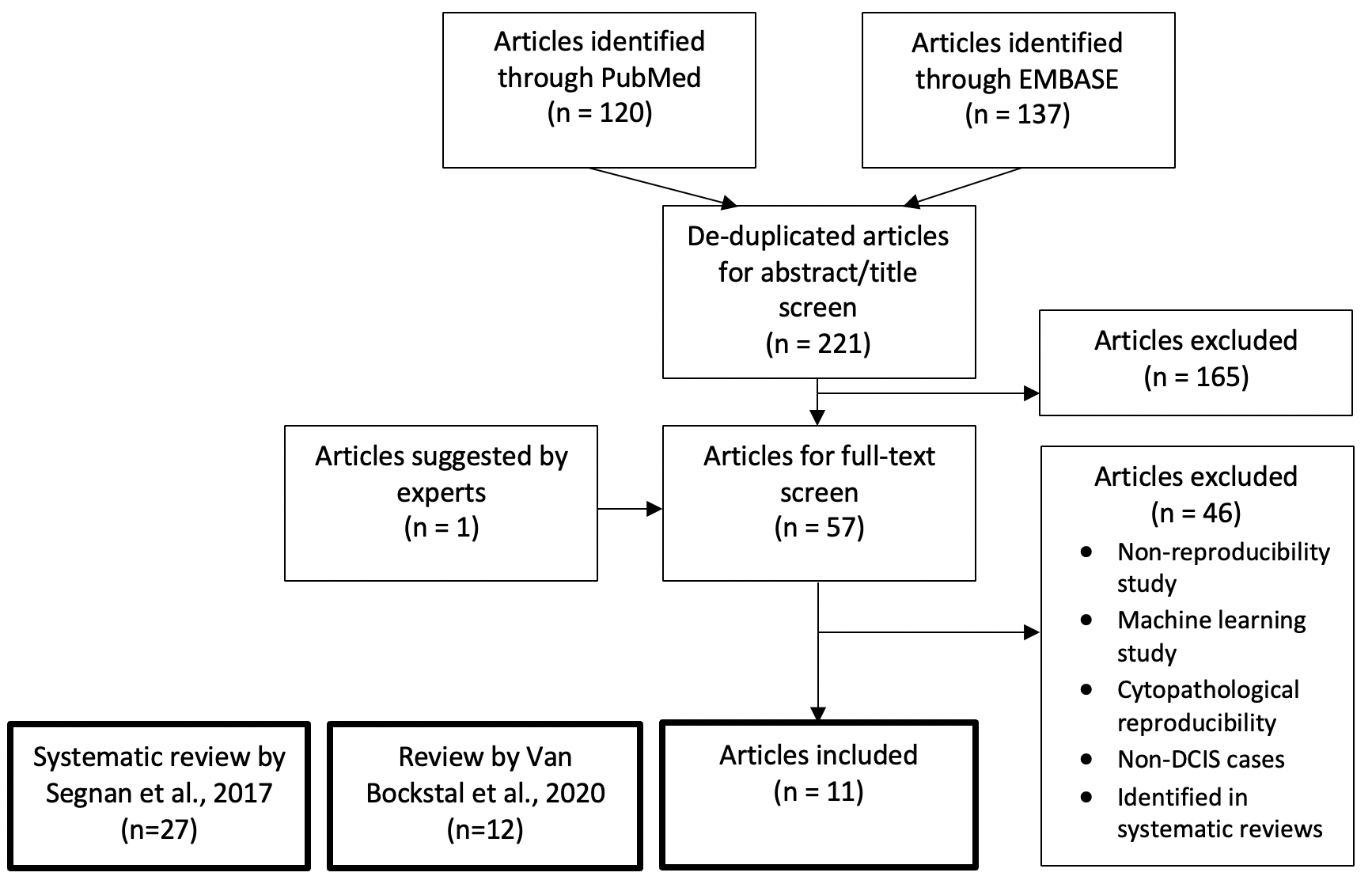


2d. Diagnostic drift


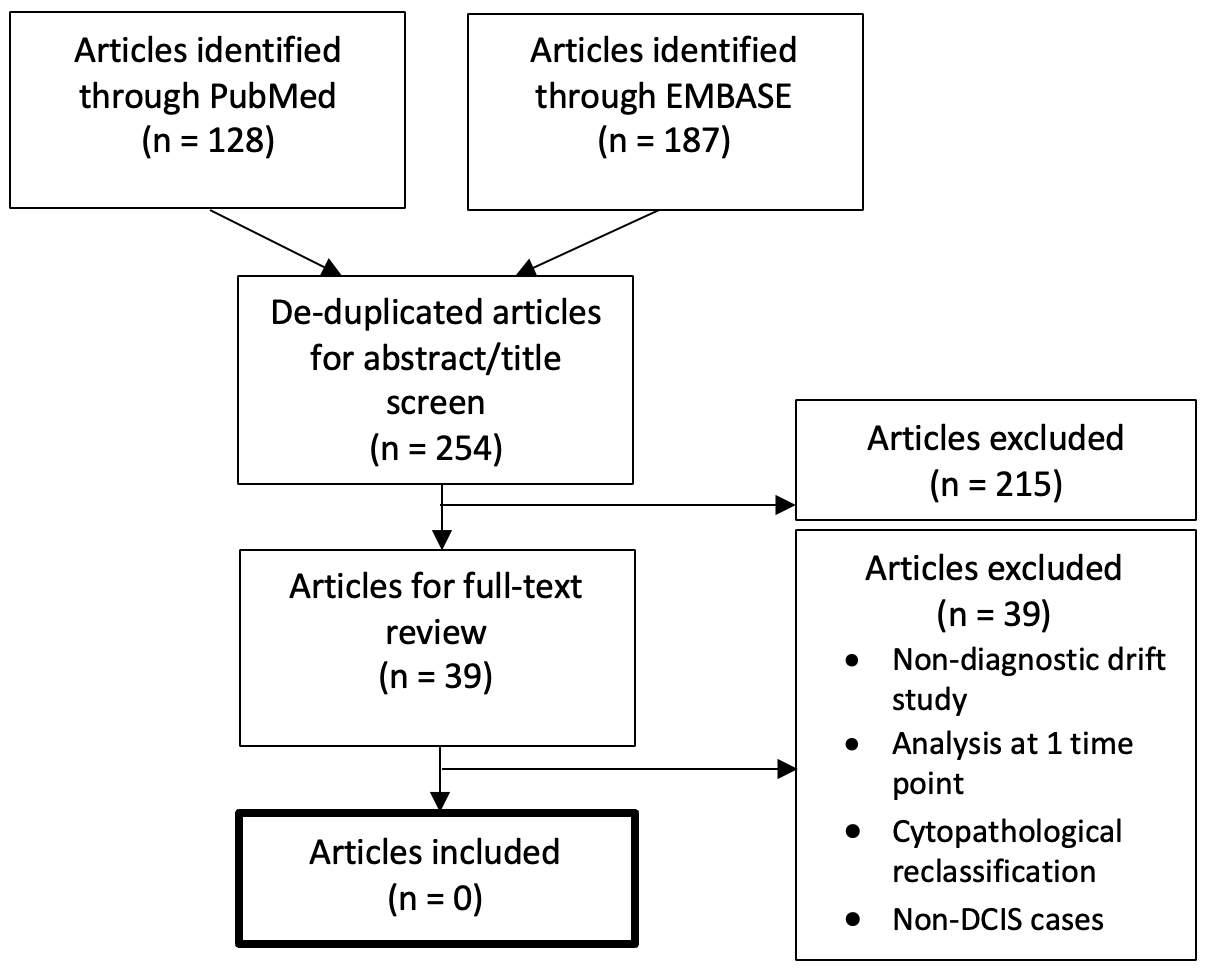


Online Appendix 3: Risk of bias assessment

**Criteria used to assess overall risk of bias for all studies:**

Low overall risk of bias: where the study has low risk of bias for all domains.

Moderate overall risk of bias: where the study fails the low overall risk criteria, but does not have high risk of bias in any domain.

High overall risk of bias: where the study has high risk of bias in any domain.

3a. Natural history

|  | Domain 1 | Domain 2 | Domain 3 | Domain 4 | Domain 5 | Domain 6 | Domain 7 | Overall |
| --- | --- | --- | --- | --- | --- | --- | --- | --- |
| Akushevich et al. | **High** | **Low** | **Low** | **High** | **Low** | **Low** | **Unclear** | **High** |
| Byng et al. | **High** | **Low** | **Low** | **High** | **Low** | **Low** | **Unclear** | **High** |
| Co et al. | **High** | **Low** | **Low** | **Low** | **Low** | **Low** | **Unclear** | **High** |
| Grimm et al. | **High** | **Low** | **Low** | **High** | **High** | **Low** | **High** | **High** |
| Mannu et al. | **High** | **Low** | **Low** | **Low** | **Low** | **Low** | **Unclear** | **High** |
| Maxwell et al. | **High** | **Low** | **Low** | **High** | **High** | **Unclear** | **High** | **High** |
| Meyerson et al. | **High** | **High** | **Low** | **Low** | **Low** | **Low** | **High** | **High** |
| Ryser et al. | **High** | **Low** | **Low** | **High** | **Low** | **Low** | **Unclear** | **High** |
| Sagara et al. | **High** | **Low** | **Low** | **High** | **Low** | **Low** | **Unclear** | **High** |

Domain 1: Baseline and/or time-varying confounders

Domain 2: Selection bias

Domain 3: Classification of interventions

Domain 4: Deviation from intended interventions

Domain 5: Non-availability of data

Domain 6: Measurement bias

Domain 7: Reporting bias

3b. Reproducibility

|  | Domain 1 | Domain 2 | Domain 3 | Domain 4 | Overall |
| --- | --- | --- | --- | --- | --- |
| Brunye et al. | **Unclear** | **Low** | **Low** | **Low** | **Moderate** |
| Jackson et al. | **Low** | **Low** | **Low** | **Low** | **Low** |
| Mercan et al. | **Low** | **Low** | **Low** | **Low** | **Low** |
| Onega et al. | **Low** | **Low** | **Low** | **Low** | **Low** |
| Qiu et al. | **Unclear** | **Low** | **Low** | **Low** | **Moderate** |
| Rakha et al. (2017) | **Moderate** | **Low** | **Low** | **Low** | **Moderate** |
| Rakha et al. (2016) | **Moderate** | **Low** | **Low** | **Low** | **Moderate** |
| Tozbikian et al. | **Low** | **Low** | **Low** | **Low** | **Low** |
| Trocchi et al. | **Unclear** | **Low** | **Low** | **Low** | **Moderate** |
| Tsuda et al. | **Unclear** | **Unclear** | **Low** | **Low** | **Moderate** |
| Van Seijen et al. | **Unclear** | **Low** | **Low** | **Low** | **Moderate** |

Domain 1: Participants representative of a population sample

Domain 2: Pathologists blinded to previous diagnoses of lesions

Domain 3: Appropriate diagnostic criteria and application

Domain 4: Appropriate measures of statistical agreement used
